# Maternal Gliadin Intake Reduces Oocyte Quality with Chromosomal Aberrations and Increases Embryonic Lethality through Oxidative Stress in a *Caenorhabditis elegans* Model

**DOI:** 10.3390/nu14245403

**Published:** 2022-12-19

**Authors:** Jae Hyuck Lee, Mijin Lee, Hyemin Min, Esther Youn, Yhong-Hee Shim

**Affiliations:** Department of Bioscience and Biotechnology, Konkuk University, Seoul 05029, Republic of Korea

**Keywords:** *Caenorhabditis elegans*, gliadin, oocyte quality, oxidative stress, reproductive capacity

## Abstract

Oocyte quality is essential for reproductive capacity, but it rapidly declines with age. In addition to aging, maternal nutrition is a major concern in maintaining oocyte quality. Gliadin, a major component of gluten, causes gluten toxicity, which has been reported in a variety of gluten-related disorders. The basis of gluten toxicity in reproduction is being understood using simple animal models such as *Caenorhabditis elegans*. In this study, we examined the effects of gliadin peptide (GP; amino acids 151–170) intake on oocyte quality control in *C. elegans*. We found that GP intake impaired oocyte quality through chromosomal aberrations and mitochondrial oxidative stress, which was suppressed by antioxidant treatment. The reduced oocyte quality by GP intake consequently increased embryonic lethality. Furthermore, the expression of oxidative stress-responding genes *prdx-3* and *gst-4* was significantly increased by GP intake. The increased DAF-16 activity by GP intake suggests that DAF-16 is a possible transactivator of these antioxidant genes. Taken together, GP intake reduced reproductive capacity in *C. elegans* by decreasing oocyte quality and increasing embryonic lethality through mitochondrial oxidative stress.

## 1. Introduction

The transfer of genetic material to the next generation is an essential life activity. Germline cells play a central role in this process. Therefore, determining the regulatory mechanisms underlying the maintenance of healthy germ cells in animals is pivotal. With the increasing human life span, maternal age is increasing, resulting in reduced oocyte quality at the time of onset of fertilization [1,2]. Maternal nutrition is also a major factor in the regulation of oocyte quality [3]. Therefore, understanding not only the intrinsic, but also the extrinsic factors that modulate oocyte quality during aging is essential. Our previous studies indicated that gliadin intake increases germ cell apoptosis [4]. Germ cell apoptosis is a key process in maintaining oocyte quality in *Caenorhabditis elegans* [5]. Accumulating data indicate that gluten toxicity causes not only celiac, but also non-celiac diseases [6], and the choice of food is strongly associated with intergenerational effects [3]. To investigate this sophisticated challenge, a simple, but comparable, animal model is required. 

*Caenorhabditis elegans* is an excellent model organism for studying the effects of nutrients on germ cell development because its somatic cell development is mostly complete, while germ cell development is highly active at the adult stage, and its life cycle is short and distinct [7,8]. The embryo undergoes embryogenesis for 10–12 h, and the hatched embryo at larval stage 1 passes larval stages 2, 3, and 4 and becomes a day-1 adult within 65 h at 20 °C [9]. When worms become day-3 adults, reproductive aging begins, followed by somatic aging after day 5 and keeps aging until death on day 15 [10,11]. *Caenorhabditis elegans* exists as both a hermaphrodite and a male. In the hermaphrodite germline, germ cell developmental processes such as mitosis, meiosis, spermatogenesis, and oogenesis can be observed, proving to be advantageous for studying germ cells at different stages during development [8,12]. In addition, since the intestine is closely positioned to the gonad, inter-tissue interactions between them are more likely to occur. Indeed, we previously found that reproductive capacity in *C. elegans* is influenced by intestinal barrier disruption caused by gliadin intake [4].

Gluten is composed of gliadin and glutenin, a major component of wheat [13]. Undigested gliadin causes gluten toxicity [13]. Since ancient times, wheat has been a major food source and its consumption is steadily increasing [14]. Recently, an increased number of people are suffering from gluten-related diseases, including defects in fertility [15]. We have previously found that gliadin intake induces oxidative stress in *C. elegans* [16]. Furthermore, oxidative stress generated by an imbalance in redox homeostasis leads to poor oocyte quality [17]. These findings suggest that gliadin intake is a harmful extrinsic factor for the reproductive capacity of *C. elegans*, which is a good model for examining the effects of maternal nutrition on oocyte quality control. Therefore, in this study, we examined the adverse effects of gliadin intake on oocytes and, consequently, on offspring. Based on our results, maternal gliadin intake reduces oocyte quality and exerts intergenerational effects through mitochondrial oxidative stress.

## 2. Materials and Methods

### 2.1. Caenorhabditis elegans Strains and Gliadin Peptide (GP) Treatment 

*Caenorhabditis elegans* strains were maintained at 20 °C on nematode growth medium (NGM) agar plates containing *Escherichia coli* strain OP50. N2 (*C. elegans* wild isolate, Bristol), ERT60: *jyIs13 (act-5p::GFP::ACT-5 + rol-6(su1006)) II*, and GR1352: *xrIs87 (daf-16(alpha)::GFP::daf-16B + rol-6(su1006))* were used. A synthetic GP containing amino acids 151–170 of gliadin was commercially synthesized and labeled with FITC from ANYGEN (ANYGEN, Gwangju, Korea) and used at a final concentration of 3 µM, as previously described [4]. Synchronized L4-stage larvae were treated with GP for 24 h (day-1 adult) or 72 h (day-3 adult) at 20 °C, and each group of worms was examined.

### 2.2. Mitochondrial Reactive Oxygen Species (mtROS) Analysis and N-Acetyl-L-Cysteine (NAC) Treatment

CellROX^®^Green (Invitrogen, Carlsbad, CA, USA) staining was used to detect mtROS levels in GP-treated worms. The worms were incubated in NGM agar plates containing 5 µM CellROX^®^Green dye for 1 h at 20 °C, and the CellROX^®^Green signal was observed. Dissected gonads and oocytes were also analyzed by dissecting worms in 5 µM CellROX^®^Green dye on a poly L-lysine-coated slide and incubated in a wet chamber for 30 min at 20 °C. The CellROX^®^Green signal was observed under a fluorescence microscope (Zeiss Axioscope, Oberkochen, Germany), and its intensity was quantified using ImageJ software (v.1.53e software, National Institute of Health, Bethesda, MD, USA). NAC (Sigma-Aldrich, St. Louis, MO, USA) was used to examine the antioxidant effect. Worms fed with GP were treated with the antioxidant NAC. Synchronized L4-stage worms were placed on NGM plates containing 0 or 3 µM GP for 48 h at 20 °C, and then the worms were transferred to 0 or 5 mM NAC-treated NGM plates containing 0 or 3 µM GP for 24 h at 20 °C.

### 2.3. DNA Staining in Oocytes, Embryonic Lethality, and Survival Assay

Worm gonads were dissected in 0.2 mM tetramisole hydrochloride (Sigma-Aldrich, St. Louis, MO, USA) on a poly L-lysine-coated slide and fixed with cold methanol and acetone. The extruded gonads containing oocytes were stained in Vectashield with DAPI (4’,6-diamidino-2-phenylindole, Vector Laboratories, Burlingame, CA, USA), and the chromosomal morphology in the oocytes was observed under a fluorescence microscope (Zeiss Axioscope, Oberkochen, Germany). Embryonic lethality was measured to analyze the effects of maternal GP intake on oocyte quality. It was calculated as the percentage of non-hatched embryos out of the total number of progenies produced by day-3 adults in 24 h. Non-hatched embryos after 24 h of incubation were considered dead.

Wild-type N2 worms were treated with 0 or 3 µM GP at the L4 stage for 3 days. The day-3 adults laid eggs for 24 h, and the hatched L1-staged F1 progeny were incubated in M9 buffer containing 100 mM paraquat (1,1’-dimethyl-4,4’-bipyridinium dichloride, Sigma-Aldrich, St. Louis, MO, USA) for 6 h at 20 °C. The surviving worms were counted. Worms were considered dead when they did not respond to gentle touch with a platinum wire.

### 2.4. Quantitative Reverse Transcription-Polymerase Chain Reaction (qRT-PCR)

The total RNA was extracted from the worms using TRIzol reagent (Invitrogen, Waltham, MA, USA), and the RNA was isolated using a phase-lock gel (MaXtract High Density; Qiagen, Germantown, MD, USA) as previously described [18]. *cdc-42* was used as an endogenous control for normalization, and the relative expression of each gene was calculated compared with that of the control. All experiments were independently performed in triplicate. The primers used were as presented in the Supplementary Table S1. 

### 2.5. Live Image Observation of Fluorescence-Tagged Transgenic Animals

Synchronized L4-stage larvae were treated with 0 or 3 µM GP for 72 h at 20 °C. The worms were then immobilized using a 0.2 mM tetramisole hydrochloride (Sigma-Aldrich, St. Louis, MO, USA) on a poly L-lysine-coated slide. Live images of the worms were observed under a fluorescence microscope (Zeiss Axioscope, Oberkochen, Germany).

### 2.6. Intestinal Barrier Function Assay

To observe the intestinal barrier function, the worms were incubated for 3 h in liquid cultures of OP50 bacteria mixed with blue food dye (FD&C Blue No.1 FD110, Spectrum, New Brunswick, NJ, USA) at 20 °C. The worms were then transferred to NGM plates seeded with OP50. The presence of blue food dye in the body cavity indicated intestinal leakage.

### 2.7. Statistical Analysis

Statistical analyses were performed using Jamovi software (https://www.jamovi.org/, accessed on 30 December 2021). The *p*-values were evaluated using Student’s *t*-test, two-way analysis of variance (ANOVA) with Tukey’s post hoc test, or chi-square test. Data are presented as mean ± standard deviation (SD). Statistical significance was set at *p* < 0.05. All experiments were performed more than three times for statistical data calculation.

## 3. Results

### 3.1. Mitochondrial Reactive Oxygen Species (mtROS) Production Was Increased in Gliadin Peptide (GP)-Treated Adult C. elegans

**Figure 1:**
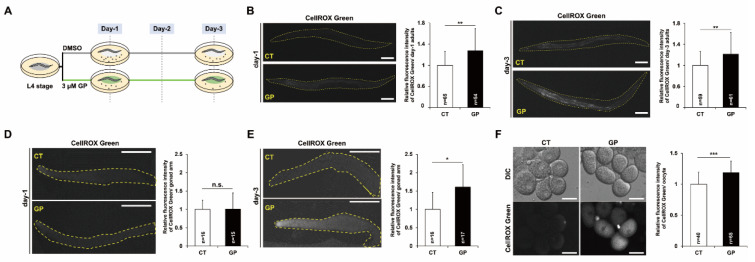
Gliadin peptide (GP) intake increases mitochondrial ROS production (mtROS) in adult *C. elegans*. (**A**) Experimental scheme of GP treatment. The synchronized wild-type N2 worms at L4 stage were incubated on NGM plates containing 0 (DMSO) or 3 µM of GP at 20 °C, and then worms were examined at day 1 or day 3 post L4 stage. (**B**–**F**) Comparison of the mtROS levels in worms (**B**,**C**), in the dissected gonad (**D**,**E**), or in the oocytes (**F**) treated with 0 (CT) and 3 µM of GP at the L4 stage for 1 day (**B**,**D**) or 3 days (**C**,**E**,**F**) using CellROX^®^Green staining. The dashed line indicates the outline of the whole body (**B**,**C**) or distal gonad (**D**,**E**). Scale bars are 50 µm (**B**–**E**) or 20 μm (**F**). Error bars represent SD. n.s.: not significant, * *p* < 0.05, ** *p* < 0.005, *** *p* < 0.001 (Student’s *t*-test).

To overcome the low penetrance of gliadin, we previously used a synthetic GP consisting of a functional motif of gliadin to examine its physiological activity in *C. elegans* [4]. GP151-170, which exhibits gut-permeating activity, showed a high induction of intracellular ROS production in both mammalian cells and *C. elegans* [4,19]. Therefore, we further examined the role of gliadin in mtROS production using GP151-170 at different developmental stages, day-1 and day-3 post L4 stage of *C. elegans* (Figure 1A). 

We performed CellROX^®^Green staining, using a fluorescent dye that detects mtROS by binding to oxidatively damaged mtDNA. Both the soma and germline of adult *C. elegans* were examined. To examine the germline, the gonad was extruded by dissecting the adult *C. elegans*. The mtROS levels were significantly increased in soma on both day-1 (Figure 1B) and day-3 (Figure 1C) in adult *C. elegans* with GP intake. However, the mtROS levels were not changed with GP intake in the dissected germline on day-1 (Figure 1D), whereas it was significantly increased in the day-3 adult germline (Figure 1E). These results suggested that the effect of GP intake on the induction of mitochondrial oxidative stress in the germline was augmented in day-3 adult *C. elegans*. We further examined the mtROS levels induced by GP intake in the oocytes of day-3 adult *C. elegans*. Notably, mtROS production significantly increased in the oocytes produced from GP-treated day-3 adults (Figure 1F). Taken together, these findings suggest that GP intake increases the mtROS levels in the germline and oocytes with age. 

### 3.2. Oocyte Quality, Embryonic Lethality, and Oxidative Stress Sensitivity in GP-Treated Adult C. elegans and Offspring

Our previous studies indicated that gliadin intake increases germ cell apoptosis [4]. Germ cell apoptosis in *C. elegans* is an important process for maintaining oocyte quality [5]. In addition, oxidative stress in oocytes that undergo meiotic prophase accelerates chromosome missegregation, resulting in low-quality oocytes [17]. Therefore, we examined the quality of oocytes produced by GP-treated adult *C. elegans*. We examined -1 oocytes (most proximal oocytes) of day-3 adults using DNA staining. During oogenesis, mature oocytes in *C. elegans* are in meiotic prophase I before fertilization and have six pairs of aligned and condensed chromosomes [20].

**Figure 2:**
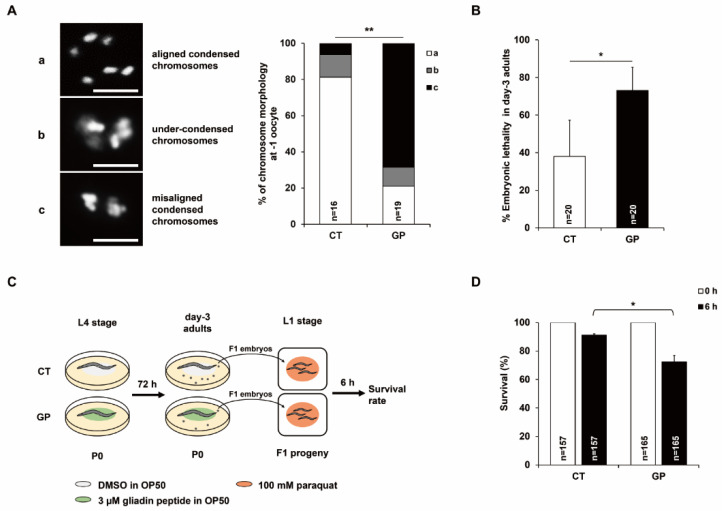
Chromosomal aberrations in the oocytes, embryonic lethality, and oxidative stress sensitivity in offspring are increased by GP intake. (**A**) Chromosomal morphology was observed using DNA staining. The type of aberration was classified into three categories depending on the condensation of chromosomes: aligned and condensed (a), under-condensed (b), and misaligned and condensed (c). The graph indicates the chromosomal morphology of -1 oocytes produced from control (CT) or GP-treated adult worms. Scale bars are 10 μm. ** *p* < 0.05 (chi-square test). (**B**) The graph indicates the percentages of unhatched embryos for 24 h in day-3 adult-stage worms treated with 0 (CT) or 3 µM of GP. Error bars represent SD. * *p* < 0.05 (Student’s *t*-test). (**C**) Experimental scheme for survival assay against 100 mM paraquat (PQ) in the offspring produced from DMSO-treated (CT) or GP-treated worms (GP). (**D**) The graph indicates survival rate in the F1 progenies produced by worms treated with 0 (CT) or 3 µM GP and 100 mM PQ for 6 h. Error bars represent SD. * *p* < 0.05 (two-way ANOVA with Tukey’s post hoc test).

Chromosomal aberrations in the -1 oocytes were categorized into three classes: aligned and condensed (normal six-bivalent), under-condensed, and misaligned and condensed (Figure 2A). Chromosomal aberrations (under-condensed, misaligned, and condensed chromosomes) were observed in the -1 oocytes of GP-treated day-3 adults, and defective -1 oocytes increased with GP intake (Figure 2A). To determine whether increased chromosomal aberrations lead to embryonic lethality, we measured embryonic lethality in offspring of GP-treated day-3 adult mothers. Embryonic lethality was significantly increased in GP-treated day-3 adult worms compared with that in the control (Figure 2B). These results indicate that oocyte quality was reduced by chromosomal aberrations in GP-treated worms, resulting in increased embryonic lethality. Based on these findings, we hypothesized that the increased levels of mtROS and chromosomal aberrations in oocytes influence the oxidative stress response in embryos and subsequently F1 progeny. To test this hypothesis, we measured the survival rate of F1 progeny upon exposure to 100 mM paraquat (PQ, a ROS generator) for 6 h (Figure 2C). We found that the progeny from GP-fed worms exhibited a decreased survival rate compared with that of the progeny from worms non-treated with GP (Figure 2D). This result suggests that the progeny from GP-fed worms have decreased oxidative stress resistance. 

### 3.3. N-Acetyl-L-Cysteine (NAC) Treatment Suppresses Adverse Effects of GP Intake

**Figure 3:**
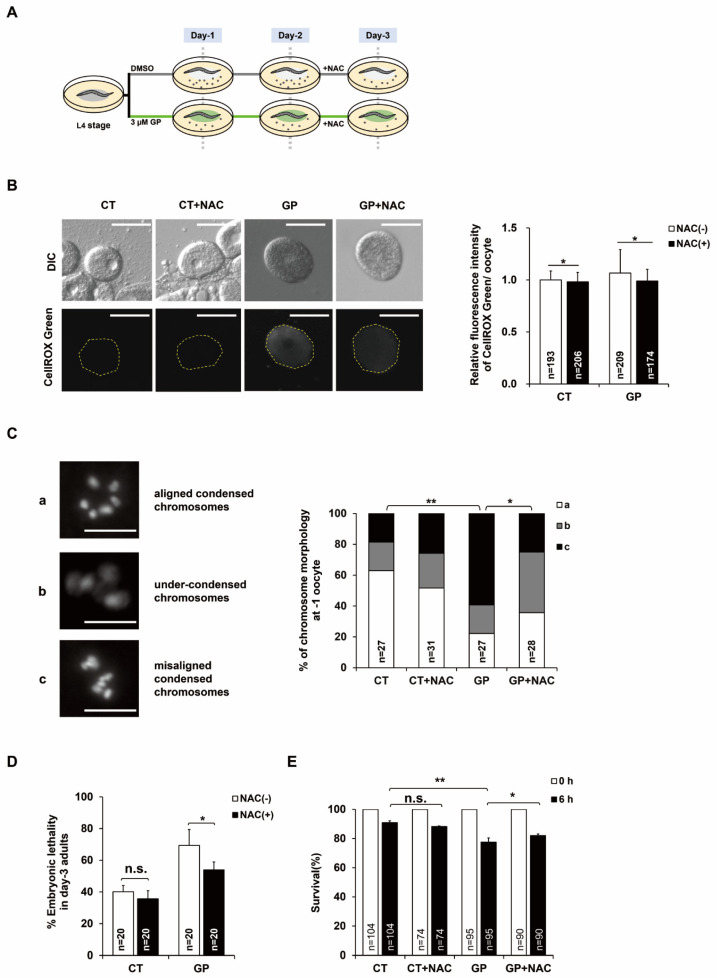
N-acetyl-L-cysteine (NAC) treatment suppresses effects of GP intake in adult *C. elegans*. (**A**) Experimental scheme of NAC treatment. The synchronized wild-type N2 worms at L4 stage were incubated on NGM plates containing 0 (DMSO) or 3 µM of GP at 20 °C, and treated with NAC for 24 h, and then worms were examined at day 3 post L4 stage. (**B**) Comparison of mtROS levels in oocytes produced from control (CT) and GP-treated adult worms without (CT, GP) or with NAC (CT + NAC, GP + NAC) treatment using CellROX^®^Green staining. The bar graph indicates the relative pixel intensity of fluorescence of CellROX^®^Green. Scale bars are 20 μm. Error bars represent SD. * *p* < 0.05 (Student’s *t*-test). (**C**) The graph indicates chromosomal morphology of -1 oocytes produced from control (CT) or GP-treated adult worms without (CT, GP) or with NAC (CT + NAC, GP + NAC) treatment using DNA staining. The types of aberrations were classified into three categories depending on the chromosomal morphology: aligned and condensed (a), under-condensed (b), and misaligned and condensed (c). Scale bars are 10 µm, * *p* < 0.05, ** *p* < 0.005 (chi-square test). (**D**) The graph indicates the percentages of unhatched embryos for 24 h in day-3 adult worms treated with 0 (CT) or 3 µM of GP with or without NAC. Error bars represent SD. n.s.: not significant. * *p* < 0.05 (Student’s *t*-test). (**E**) The graph indicates survival rate in the F1 progenies produced by 0 (CT) or 3 µM of GP-treated worms without (CT, GP) or with NAC (CT + NAC, GP + NAC) after 100 mM paraquat treatment for 6 h. Error bars represent SD. n.s.: not significant. * *p* < 0.05, ** *p* < 0.005 (two-way ANOVA with Tukey’s post hoc test).

To confirm whether the toxic effects of GP intake on oocyte quality in the germline of adult *C. elegans* are due to oxidative stress, the antioxidant NAC was used, and the mtROS production and chromosomal morphology of the oocytes produced by GP-treated or GP-NAC-treated day-3 adult *C. elegans* were examined. The GP-treated day-2 adults were treated with NAC for 24 h at 20 °C, and the oocytes were isolated from GP-treated or GP-NAC-treated day-3 adults and the mtROS levels were measured (Figure 3A). The mtROS levels in oocytes produced by GP-treated worms were decreased by NAC treatment (Figure 3B). We further examined the chromosomal morphology of the oocytes (Figure 3C). As hypothesized, the chromosomal aberrations in the oocytes of GP-treated worms were reduced by NAC treatment, showing an increased proportion of type a, normal type, and a decreased proportion of severely abnormal type c (Figure 3C). These results suggest that the chromosomal aberrations caused by GP intake were indeed attributed to oxidative stress, owing to the increased mtROS production in the oocytes produced by day-3 adult *C. elegans*. Similarly, we confirmed that NAC treatment decreased the embryonic lethality induced by GP intake in day-3 mothers (Figure 3D). Further, NAC treatment increased oxidative stress resistance in the F1 progeny of GP-treated worms, as indicated by an increased survival rate (Figure 3E). These results suggest that GP intake reduces oocyte quality and increases embryonic lethality via oxidative stress, which can be suppressed by NAC treatment.

### 3.4. Expression of Oxidative Stress-Responding Genes Increased with GP Intake 

Since GP intake exhibited toxic effects on oocyte quality by inducing mitochondrial oxidative stress, we examined the expression of oxidative stress-responding genes, including *sod* (superoxide dismutase), *ctl* (catalase), *prdx* (peroxiredoxin), and *gst* (glutathione S-transferase), using qRT-PCR in day-3 adults. The products of these genes function as antioxidants in *C. elegans* [21].

**Figure 4:**
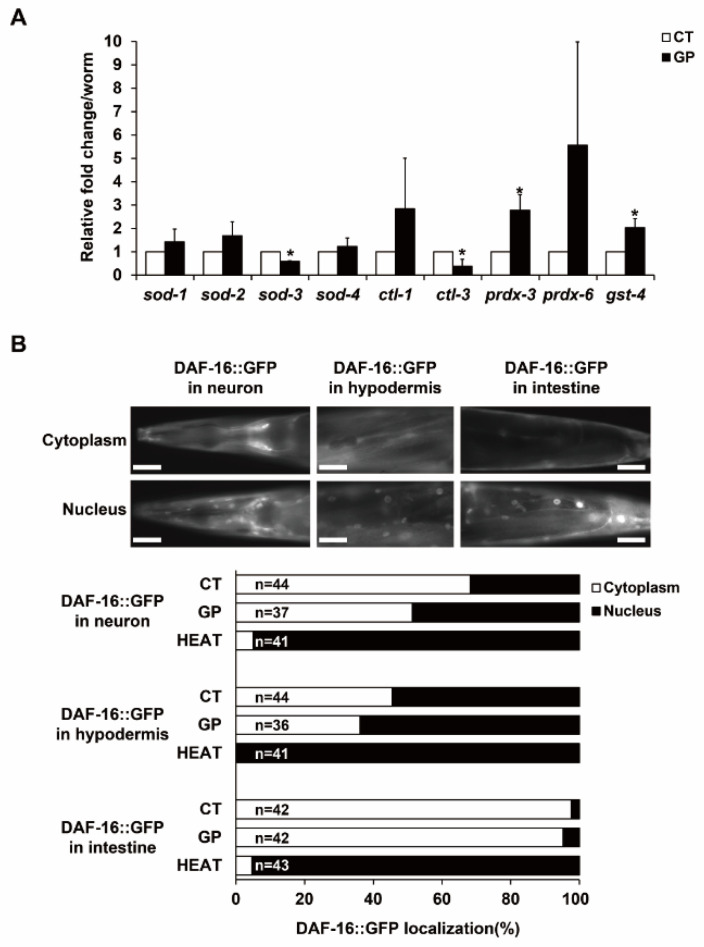
GP intake regulates the expression of antioxidant genes and DAF-16 activity in adult *C. elegans*. (**A**) The mRNA levels of antioxidant genes in GP-treated adult *C. elegans* were detected by three independent quantitative reverse transcription-polymerase chain reaction analyses using *cdc-42* mRNA in each sample as an internal control for normalization. Error bars represent SD. * *p* < 0.05 (Student’s *t*-test). (**B**) Nuclear localization of DAF-16::GFP was observed in worms treated without (CT) or with GP. The graph indicates the percentage of nuclear localization of DAF-16::GFP in neurons, hypodermis, and intestines. HEAT indicates heat shock at 37 °C for 15 min, which is used for the positive control of nuclear localization of DAF-16::GFP. Scale bars are 50 µm.

GP intake also increased the expression of most of the test genes, except *sod-3* and *ctl-3*, which remove superoxide radicals and hydrogen peroxide in mitochondria, respectively [22,23] (Figure 4A). The mRNA levels of *prdx-3* and *gst-4*, which encode for major cellular detoxification enzymes that maintain redox homeostasis in *C. elegans* [23,24], were significantly increased by more than two-fold in GP-treated worms (Figure 4A). In addition, GP intake increased mRNA levels of *ctl-1* and *prdx-6*. These results suggest that antioxidant genes respond to GP intake in a gene-specific manner via PRDX-3 and GST-4. We further examined the possible transcriptional activators of *prdx-3* and *gst-4* by investigating the nuclear localization of DAF-16 in transgenic worms expressing a *daf-16::GFP* transgene (Figure 4B). DAF-16, a forkhead box O (FOXO) transcription factor in *C. elegans*, transcriptionally regulates antioxidant genes in response to oxidative stress [25,26,27] and is activated by nuclear localization [28]. Notably, although it did not show as marked a response as it did to heat treatment, the proportion of nuclear-localized DAF-16 in all three tissues, including neurons, hypodermis, and intestine, increased in response to GP intake (Figure 4B). This suggests that GP intake modulates DAF-16 activity, which, in part, transcriptionally regulates mitochondrial oxidative stress responses in *C. elegans*.

### 3.5. GP Intake Causes Disruption of Intestinal Barrier in Adult C. elegans

Oxidative stress through ROS production is associated with many intestinal diseases causing intestinal barrier damage in mice [29]. In our previous study, we showed that gliadin intake induced intestinal barrier dysfunction through ROS production in *C. elegans* [4,16]. Therefore, we also examined whether GP intake affected intestinal leakage using blue food dye staining in day-3 adult worms. 

**Figure 5:**
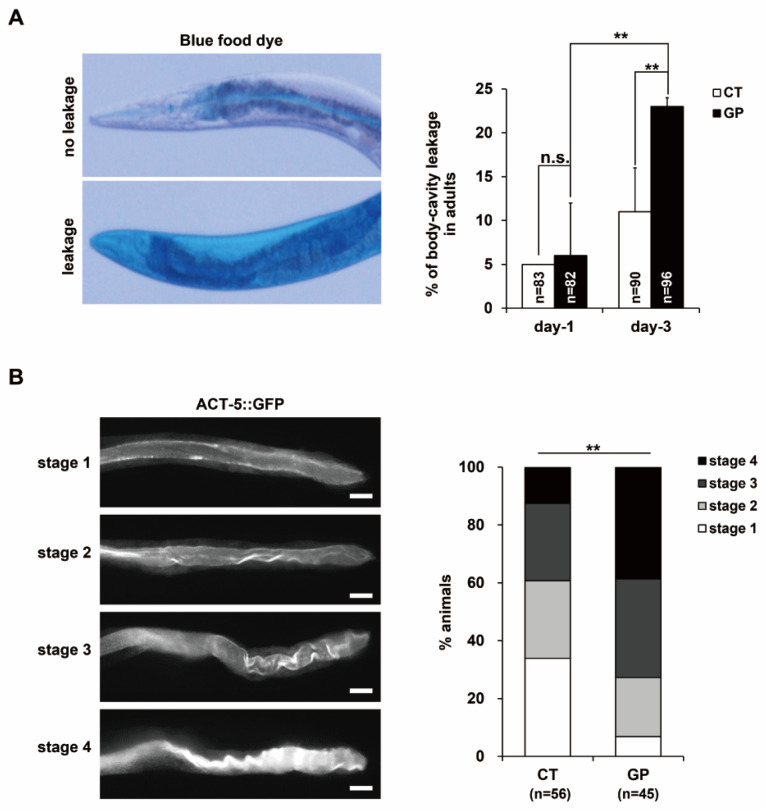
GP intake promotes intestinal disruption in adult *C. elegans*. (**A**) The presence of blue food dye in the body cavity indicates leakage of the intestinal barrier after 0 (CT) or 3 µM of GP treatment. Error bars represent SD. n.s.: not significant. ** *p* < 0.05 (chi-square test). (**B**) Transgenic worms expressing *actin 5 (ACT-5)::GFP* were treated with 0 (CT) or 3 µM of GP at the L4 stage for 3 days. The intestinal actin localization was classified into four stages depending on mislocalization in the apical side of the intestine. The percent distributions of the respective stage in worms are presented in the graph. Scale bars are 50 µm, ** *p* < 0.05 (chi-square test).

Intestinal leakage was significantly increased in day-3 adult worms with GP intake compared with that of the control without GP and to that of day-1 adult worms with GP treatment (Figure 5A), suggesting that GP intake promotes intestinal leakage in *C. elegans* and its effect is augmented with age. We confirmed the disrupted intestinal integrity using transgenic worms, ERT60, which contain a transgene *act-5::GFP*. ACT-5 is a major cytoskeleton component involved in intestinal maintenance in *C. elegans* [30]. Therefore, ACT-5 mislocalization indicates disrupted intestinal integrity. Consistent with the above observations, the GP-treated worms exhibited more severely mislocalized ACT-5 compared with that of the control (Figure 5B). Taken together, these results indicate that GP intake impairs intestinal integrity in *C. elegans* with age and subsequently influences germ cell quality.

## 4. Discussion

The maintenance of oocyte quality is essential for producing healthy offspring [31,32]. As reproductive aging begins, oocyte quality rapidly declines [10]. At this stage of development, germ cells are highly vulnerable to environmental factors including maternal nutrition [31,33]. In this study, we attempted to elucidate the effects of maternal gliadin intake on oocyte quality and embryonic development. We analyzed the changes in oocytes and subsequent offspring in response to the oxidative stress induced by maternal GP intake in *C. elegans*. 

Taking advantage of the well-defined *C. elegans* developmental stages, we clearly analyzed day-1 and day-3 adults and compared their responses to GP intake. Both day-1 and day-3 adults are somatically healthy; however, their reproductive capacity begins to decline from day 3 [10,11]. Therefore, we hypothesized that the toxic effects of GP intake on reproduction is augmented in day-3 adults, and day 3 is an appropriate time to analyze the effects of maternal GP intake on reproductive capacity. Indeed, GP intake significantly increased oxidative stress in the oocytes and intestine of day-3 adults. Considering that the intestine is healthy, but the germline is declining at this stage, the findings suggest that GP intake accelerates intestinal and reproductive aging. Notably, severe changes were observed due to GP intake in germ cells from day-3 adults, whereas only a small change was observed in those from day-1 adults, suggesting that the impact of maternal GP intake is more severe in reproductively aged oocytes. This result indicates that the effects of GP depend on age. The age-dependent effect on oocytes may be associated with that in *C. elegans*; oocyte quality control decreases in day-3 adults [34,35]. Oocyte quality control in *C. elegans* is mainly performed by germ cell apoptosis [5]. Further, germ cell apoptosis is gradually elevated at the day-1 and day-2 adult stages when the oocyte is abundantly produced and thereafter, begins to decrease [5,10]. This may support the finding that only a small change was observed in the oocytes of day-1 adults. 

In *C. elegans*, the oxidative stress induced by environmental factors deteriorates fertility, and this effect can be reversed by antioxidant treatment [36]. Similarly, GP-treated day-3 adults exhibited increased intestinal leakage and mtROS production in both the intestine and oocytes. These effects were suppressed by NAC treatment. Excessive ROS levels cause damage to cellular components [37]. These findings indicate that the toxic effects of GP intake are indeed due to oxidative stress and disrupted intestinal integrity, which is linked to alterations in oocytes, including chromosomal aberrations. Defective oocytes with chromosomal abnormalities eventually increased embryonic lethality, and some surviving offspring developed from these oocytes exhibited decreased oxidative stress resistance. Defective oocytes with chromosomal aberrations are known to have impaired embryonic development [38]. These findings imply that GP intake causes oxidative stress, resulting in a decline in oocyte quality and, subsequently, unhealthy offspring. In addition, we found that GP intake promoted DAF-16 translocation and the expression of its downstream targets, such as *prdx-3* and *gst-4*. *prdx-3* and *gst-4* are oxidative stress-responding genes in the mitochondria or intestine, respectively [23,26,39]. Although GP intake increased the expression of most antioxidant genes, the oxidative stress caused by GP intake remained. This suggests that antioxidant genes are activated against oxidative stress, but they cannot maintain redox homeostasis in the presence of GP. Notably, we found that GP intake significantly decreased the mRNA levels of *sod-3* and *ctl-3*, which encode for ROS-detoxifying enzymes in the mitochondria [40]. Furthermore, *ctl-3* is a germline-enriched gene [41]. These findings partly support the increase in mtROS levels caused by GP intake in the germline and oocytes.

**Figure 6:**
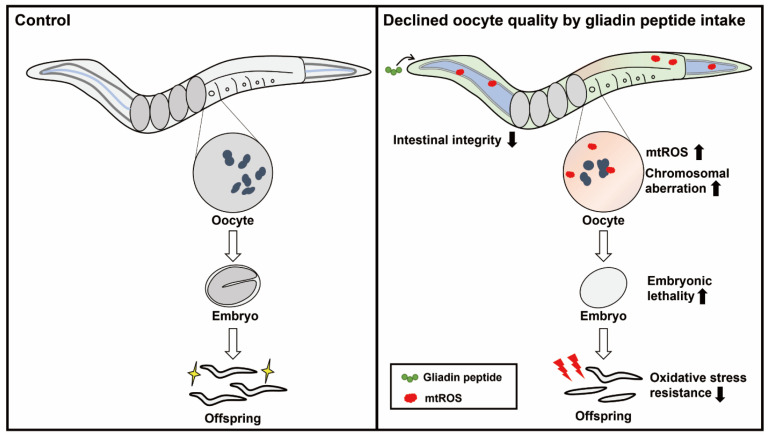
Working model: maternal gliadin peptide (GP) intake in day-3 adult *C. elegans* increases the level of mitochondrial ROS (mtROS) and decreases oocyte quality with chromosomal aberrations, consequently increasing embryonic lethality and decreasing oxidative stress resistance in offspring.

A simple model is beneficial for understanding sophisticated biological phenomena. Herein, we examined the effects of maternal nutrition on oocyte quality in *C. elegans* using GP intake. Based on our results, we suggest that maternal gliadin intake extrinsically reduces oocyte quality with age by increasing mitochondrial oxidative stress, and we propose a link between intestinal integrity and germline quality control (Figure 6).

## 5. Conclusions

This study provides several pieces of evidence showing the harmful effects of gliadin peptide (GP) intake on the quality of oocytes and their offspring by mitochondria oxidative stress and disrupted intestinal integrity in a day-3 adult *C. elegans* model. Maternal GP intake reduces oocyte quality by increasing chromosomal aberrations and embryonic lethality, and decreasing oxidative stress resistance in offspring. These findings support that maternal nutrition is strongly associated with reproductive capacity and offspring health. In this process, there is a link between the intestine and the germ cells, exerting clear inter-tissue interaction.

## Supplementary Materials

The following supporting information can be downloaded at: https://www.mdpi.com/article/10.3390/nu14245403/s1, Table S1: List of quantitative real-time PCR primers.
