# Supplementary material for: Maternal Gliadin Intake Reduces Oocyte Quality with Chromosomal Aberrations and Increases Embryonic Lethality through Oxidative Stress in a Caenorhabditis elegans Model
Source: Nutrients. 2022 Dec 19;14(24):5403. doi: 10.3390/nu14245403 (PMC9787971; doi:10.3390/nu14245403)
Supplement: Supplementary file 1 [file nutrients-14-05403-s001.zip › nutrients-2086719-supplementary.pdf]

**Supplementary Table S1. List of quantitative real-time PCR primers**

| Name | Primer sequence (5'-3') |
|---|---|
| *cdc-42* forward | 5′-AGCCATTCTGGCCGCTCTCG-3′ |
| *cdc-42* reverse | 5′-GCAACCGCTTCTCGTTTGGC-3′ |
| *ctl-1* forward | 5′-CGGATACCGTACTCGTGATGAT-3′ |
| *ctl-1* reverse | 5′-CCAAACAGCCACCCAAATCA-3′ |
| *ctl-3* forward | 5′-AGTAAATCTTCAAAATGCCAATG-3′ |
| *ctl-3* reverse | 5′-GGTGGGGTTCCTGATTTCTAT-3′ |
| *gst-4* forward | 5′-CGTTTTCTATGGAAGTGACGC-3′ |
| *gst-4* reverse | 5′-TCAGCCCAAGTCAATGAGTC-3′ |
| *sod-1* forward | 5′-ATTTCTGCCGGTCCACACTT-3′ |
| *sod-1* reverse | 5′-CCATAGATCGGCCAACGACA-3′ |
| *sod-2* forward | 5′-GAGGCGGTCTCCAAAGGAAA-3′ |
| *sod-2* reverse | 5′-GAACAGCGACAGTTGATGCC-3′ |
| *sod-3* forward | 5′-GGATGGTGGAGAACCTTCAA-3′ |
| *sod-3* reverse | 5′-AAGGATCCTGGTTTGCACAG-3′ |
| *sod-4* forward | 5′-GAAGCTTAACGGATCGGTTTCCGGA-3′ |
| *sod-4* reverse | 5′-TGCACCATGGCTCAGCTTATGAGGA-3′ |
| *prdx-3* forward | 5′-GTTCCGTTCTCTTGGAGCTG-3′ |
| *prdx-3* reverse | 5′-CTTGTTGAAATCAGCGAGCA-3′ |
| *prdx-6* forward | 5′-GGAGAACAATGGCTGATGC-3′ |
| *prdx-6* reverse | 5′-ATCTGAACAT GGCGTTTGC-3′ |
